# Kinematic Loggers—Development of Rugged Sensors and Recovery Systems for Field Measurements of Stone Rolling Dynamics and Impact Accelerations during Floods

**DOI:** 10.3390/s22031013

**Published:** 2022-01-28

**Authors:** Hamish Biggs, Andrew Starr, Brendon Smith, Steve de Lima, Julian Sykes, Arman Haddadchi, Graeme Smart, Murray Hicks

**Affiliations:** National Institute of Water and Atmospheric Research (NIWA), Christchurch 8011, New Zealand; andrew.starr@niwa.co.nz (A.S.); brendon.smith@niwa.co.nz (B.S.); steve.delima@niwa.co.nz (S.d.L.); julian.sykes@niwa.co.nz (J.S.); arman.haddadchi@niwa.co.nz (A.H.); graeme.smart@niwa.co.nz (G.S.); murray.hicks@niwa.co.nz (M.H.)

**Keywords:** sediment transport, particle dynamics, IMU, accelerometer, LoRa, rugged waterproof sensors, recovery systems, environmental monitoring, rockfall dynamics, UAV

## Abstract

Discrete particle dynamics is one of the least understood aspects of river bedload transport, but in situ measurement of stone movement during floods poses a significant technical challenge. A promising approach to address this knowledge gap is to use sensors embedded within stones. Sensors must be waterproof and recoverable after being transported downstream and potentially buried by other sediment. To address this challenge rugged sensors (Kinematic Loggers) were developed for deployment inside stones (ranging in size from cobbles to boulders) during floods. The sensors feature a 9-axis inertial measurement unit, 3-axis high-g accelerometer, 128 MB flash memory, and a 433 MHz LoRa radio transmission module for sensor recovery. The sensors are enclosed in rugged waterproof housings for deployment in extreme conditions (i.e., bedload transport during floods). Novel relay units and drone-based recovery systems were also developed for finding the sensors after field deployments. Firmware to control the sensors and relay units was developed, as well as software for configuring the sensors and an android application for communicating with the sensors via the LoRa radio transmission module. This paper covers the technical development of the sensors, mounting them inside stones, and field recovery tests. Although designed for measurement of coarse bedload transport and particle dynamics during floods, the sensors are equally applicable for deployment in other harsh environments, such as to study landslide and rockfall dynamics.

## 1. Introduction

Tectonic activity, weathering, erosion, and sediment transport are key processes that shape the landscapes of the Earth [1]. In particular, sediment transport is responsible for the formation of alluvial plains and basins that provide terrain well suited to human habitation and agriculture [2,3]. The accurate quantification of sediment transport processes is crucial for predicting river geometry, gravel resources, erosion, coastal replenishment, bridge integrity, flood hazards, substrate size distributions, instream habitats, reservoir sedimentation, and river evolution following restoration [4]. Over the last two centuries many sediment transport formulae have been developed [4]. However, they are primarily empirical and often have poor predictive performance when extended to conditions outside those to which they were ‘fit’ [5,6]. This is particularly true for bedload transport formulae, where there is a scarcity of reliable field data to test them [5], as well as knowledge gaps around the dynamics of individual particles (particularly rolling dynamics) during bedload transport [5,7]. These problems are hardly surprising considering the harsh conditions at the bottom of a flooded river when gravel, cobbles, and boulders smash, roll, and grind together as they are transported downstream, and deployment of high-resolution instream monitoring equipment is extremely challenging.

Bedload transport has traditionally been measured using physical samplers [8,9], inferred from morphological change [10], estimated using acoustic methods and other surrogates [9,11], or estimated using passive tracers [12]. Measurements with physical samplers (e.g., Helley–Smith type samplers) are effective for smaller sized particles (i.e., sand and gravel), but become increasingly difficult for larger particle sizes (i.e., cobbles to boulders), for which average transport rates usually need to be quantified from sediment traps [13,14]. These measurement techniques typically focus on sediment load but miss important information on transport distances, particle dynamics, entrainment, and deposition. Passive tracer studies, using painted or compositionally-distinctive “dumb” particles [12,15], do inform on transport distances over flood events but still provide no information on instantaneous particle dynamics. An alternative approach is the use of “smart” tracers with embedded sensors, which do provide the capability to collect information about the dynamics of single stones, as well as to investigate the impact of geometry and size on their transport.

To date, field studies of streamwise “smart” stone motion have used radio-tracking [16,17,18,19,20,21,22] or GPS [23] to quantify stone displacements, rest times, and average velocities. A combination of radio-tracking (i.e., RFID tags) and accelerometers were used by [24], however the accelerometers could only measure ±3 g and had 64 kb of memory, with acceleration recorded once every 10 min. This provided a record of when stones had changed orientation (i.e., moved), but did not provide data that could be used for detailed investigation of particle dynamics. These techniques provide useful data on average stone motion and diffusion [20,25], however they are unable to resolve fine scale stone dynamics and rolling. A promising technique to resolve stone dynamics in rivers uses both embedded accelerometers and gyroscopes, making “smart-stones” [26,27,28,29]. However, design problems (e.g., measurement range, battery, memory, sensor fusion algorithms, and unit geometry) have limited the practical application of existing sensors [30], and to our knowledge there is no published data on stone rolling dynamics during floods. The use of embedded accelerometers and gyroscopes has been more successful in biomedical and sport science, such as for the quantification of ball flight dynamics [31], and in avalanche science to study rockfalls [32,33]. The development of waterproof sensors that are small enough to fit within stones (i.e., cobbles and boulders) during bedload transport in flooded rivers poses a significant challenge. This challenge is further compounded by the difficulty of recovering the instrumented stones after a flood event, since they move unpredictable distances downstream and may be underwater, or buried under deposited sediment. Thus, the development of reliable recovery systems is also crucial.

This paper aims to address these challenges, through the development of rugged sensor/logger systems (herein termed Kinematic Loggers) featuring a 9-axis inertial measurement unit for stone angular velocities, accelerations, and orientation, with a 3-axis high-g accelerometer to record shocks and impact accelerations. These sensors include a Long Range (LoRa) radio transmission module, which is used for communication with the sensors and recovery after flood events. Novel firmware was developed for the sensors featuring multiple deployment, recovery and power saving options, enabling sensor wakeup on motion and maximum functional deployment times of up to 6 months. The sensors are situated inside rugged waterproof acetal housings and are deployed inside stones that have been core drilled with a diamond hole saw. A relay unit has been developed that attaches to an unmanned aerial vehicle (drone), which is flown in a zig-zag pattern over the riverbed for recovery of the sensors based on signal strength. Field team members are then deployed to the approximate location of the sensor, where it is pinpointed using directional antennas and a metal detector. Communications with the sensors are bi-directional and a high frequency transmission mode can be triggered by team members for locating the sensors more quickly. This paper details the technical development of these sensors and recovery systems, as well as field tests of the recovery systems.

## 2. Electronics and Sensor Design

### 2.1. Overview of Kinematic Logger PCB Layout and Components

**Figure 1:**
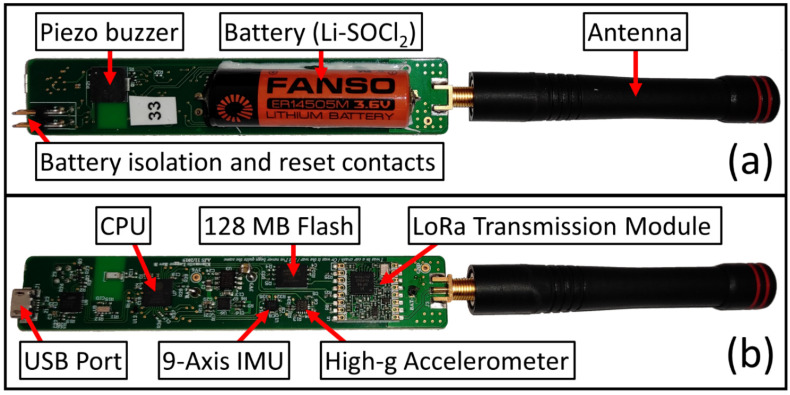
Kinematic Logger PCB, showing: (**a**) top side components, (**b**) bottom side components.

The Kinematic Logger (KL) PCB (Printed Circuit Board) and trackwork was designed in Altium 19 then manufactured by Nautech Limited. The Kinematic Logger was designed and tested in two iterations, with an overview of the final Kinematic Logger PCB components and dimensions shown in Figure 1. After development and testing there was a final production run of Kinematic Loggers, with 50 units produced in total. The Kinematic Logger PCB is 109 mm long by 20 mm wide, and the KL has a total length of 195 mm, including the antenna.

### 2.2. Components and Specifications

Central Processing Unit (CPU)

The CPU used was the Espressif ESP32-PICO-D4, which has two low-power Xtensa^®^ 32-bit LX6 240 MHz microprocessors, 4 MB of program memory, 512 KB of RAM, Wi-Fi, and Bluetooth. This CPU operates at a supply voltage between 3 V and 3.6 V, making it well suited to being powered by Lithium Thionyl Chloride Batteries (3.6 V nominal), with a linear voltage regulator (3.3 V) used to smooth the supply voltage. This CPU also features advanced control over clock speed, power modes, and wakeup interrupt timers, enabling very long deployment times with low power consumption (see Section 2.3 on power consumption and battery life). One modification that was necessary for the final version of the Kinematic Logger was the addition of two 47 μF capacitors to the input voltage supply rail (after the linear regulator) to account for short duration high current demand on CPU wakeup (inrush pulse). This was needed to account for operating the spiral wound Lithium Thionyl Chloride batteries at low temperatures (<5 °C) which they encountered overnight during field deployments in winter and spring.

Inertial Measurement Unit (IMU)

The IMU used was the 9-axis Bosch Sensortech BMX160, featuring a 3-axis accelerometer, 3-axis gyroscope and 3-axis magnetometer. This IMU is low power, accepts a wide range of input voltages (1.7–3.6 V), and can be used to send wake on motion interrupts to the CPU. The BMX160 has selectable acceleration ranges between ±16 g (16 bit) and selectable angular velocities between ±2000 °/s (16 bit). The magnetometer does not have a user-defined range but has limits of approximately ±1150 μT and resolution of 0.3 μT. The data from all channels was output at 400 Hz for post-processing and sensor fusion (see Section 2.7). The BMX160 was selected instead of the Bosch BNO055 since the BNO055 was found to be not capable of supporting a 400 Hz data rate. In addition, the BMX160 has a 16 bit (rather than 14 bit) accelerometer and uses lower power than the BNO055, making it better suited for long duration field deployments. However, the BNO055 supports absolute orientation with internal sensor fusion which may make it convenient for some deployment applications.

High-g accelerometer

To capture impact accelerations during field deployments a STMicroelectronics H3LIS331DL 3-axis high-g accelerometer was included. This accelerometer was used at an output range of ±400 g at 1000 Hz. Although the noise characteristics and accuracy of this accelerometer are poor in comparison with the BMX160, it provides an effective way to record short duration high acceleration impacts (shocks) between stones during sediment transport, which exceed the ±16 g acceleration range of the BMX160. Before sensor fusion occurs, the acceleration signals from the BMX160 and H3LIS331DL are analysed and resampled, with high-g data from the H3LIS331DL used for any timestamps where the BMX160 signal is saturated.

LoRa radio transmission module and antenna

Radio transmissions from the Kinematic Logger are sent using the LoRa (Long Range) Radio Module (HopeRF RFM98W). This has user selectable transmission power up to +20 dBm (100 mW), high receiving sensitivity down to (−144 dBm) and transmits at 433 MHz. The attenuation of radio signals in water (and other media) is frequency dependent, and 433 MHz LoRa transmitters were selected (rather than 915 MHz) to improve transmission through water (see Section 4.4). Transmission current depends on the output power (see Section 2.6) but receiving current is low (10.3 mA). To minimise current consumption, the LoRa module only listens for a user selectable time after transmitting each message packet. Each message packet sent from the Kinematic Logger consists of 6 ASCII characters, starting with ‘KL’ followed by the instrument serial number formatted as a 4-character hexadecimal string. A Linx ANT-433-CW-HD-SMA antenna was used for LoRa transmissions from the Kinematic Logger. This is a helical ¼ wavelength omni-directional whip antenna with a centre frequency of 433 MHz, peak gain of 0.7 dBi, impedance of 50 Ω and Voltage Standing Wave Ratio (VSWR) of 1.489 at 433 MHz.

Flash memory

The Kinematic Logger has a 128 MB flash memory (Winbond W25N01GVZEIG) which is used for storing the configuration settings and data from the IMU and high-g accelerometer. Data are recorded in a binary file with each record from the IMU consisting of: 1 byte for the record type, 4 bytes for the sequence number (equivalent to a timestamp), and 18 bytes for the acceleration/gyroscope/magnetometer data, with a total size of 23 bytes per record. Each record from the high-g accelerometer consists of: 1 byte for the record type, 4 bytes for the sequence number and 6 bytes for the acceleration data, with a total size of 11 bytes per record. When recording 9-axis IMU data at 400 Hz and 3-axis high-g accelerometer data at 1000 Hz, this corresponds to a data acquisition rate of 20.2 KB/s and yields a total logging time of 1 h 50 min to fill the 128 MB flash memory. However, since the Kinematic Logger is configured to sleep when not in motion, it can be deployed continuously for months on end (see Section 2.6) but will record a maximum of 1 h 50 min of motion during floods. After field deployments, data are downloaded to the computer via USB using a custom python application (see Section 2.5), then is decoded and written to a .csv file for post-processing and analysis.

Piezo buzzer

The Kinematic Logger features a piezo buzzer for debugging purposes (Murata PKMCS0909E4000-R1). This low power buzzer beeps when transitioning between logging modes and can be configured to buzz during data recording, which is useful for collecting test data. The use of a piezo buzzer, rather than a speaker with a permanent magnet (and fluctuating magnetic field during buzzing) was also advantageous to reduce magnetic field disturbances to the magnetometer in the IMU.

Real-time clock

A real-time clock (RTC) is used as the reference for logging timestamps and other activities that require the real world time. The Micro Crystal RV-3028-C7 was chosen for this role as: it has an internal oscillator minimising component count; it has an operating current of only 45 nA so has a negligible impact on battery life; it has an independent UNIX Epoch timer; and it is well-supported by third-party firmware libraries.

Voltage regulation and power management

The voltage supplied by the battery is regulated down to 3.3 V using the ABLIC S-13A1A33-E8T1U3 low dropout linear regulator. To enable auto selection of battery power or USB power, and to enable battery isolation during storage, the Linear Technology LTC4419 Dual Input Micropower PowerPath Prioritizer is also used.

USB communications

To provide USB communications for programming, configuration, and data download, the Silicon Labs CP2102N USB interface is used.

Battery

Power for the Kinematic Logger is provided by a 3.6 V Lithium Thionyl Chloride battery (AA size). These batteries have long storage shelf lives, with low self-discharge and high energy density, however their ability to deliver current depends on battery architecture and temperature. Bobbin type batteries have higher energy density but lower sustained (and pulse) current, while spiral wound batteries have lower energy density but can sustain higher current (and pulses). For the Kinematic Logger it was essential to use spiral wound batteries to deliver sufficient current for continuous logging and LoRa radio transmissions at relatively low temperatures (<5 °C). The batteries used were Fanso ER14505M which have a nominal capacity of 2200 mAh.

Battery isolation and reset

To enable batteries to be installed in advance and Kinematic Loggers to be stored ready for field deployment, a battery isolation feature was included. At the end of the Kinematic Logger (near the micro-USB port) there are two contacts for battery isolation (Figure 1). To isolate the battery a jumper cable is fit over the two contacts, then USB power is briefly applied, then finally the jumper cable is removed. There are also two contacts for system reset (Figure 1). Shorting these contacts causes a ‘soft’ system reboot.

### 2.3. Firmware and Deployment Modes

Firmware for the Kinematic Logger was written in C++ using the Espressif framework for the Arduino Integrated Development Environment (IDE). The Kinematic Logger operated in six different modes: (1) Battery isolation/shelf storage; (2) Configuration; (3) Safe; (4) Deployment delay; (5) Logging; and (6) Recovery. Battery isolation/shelf storage mode (1) is used when the Kinematic Logger will be stored for a long time before deployment without draining the battery. Configuration mode (2) is when the Kinematic Logger is plugged into a computer through the USB port and settings are written or data are downloaded. Safe mode (3) is used when data and settings should be retained on the Kinematic Logger, with it entering a very low power sleep mode and waking up every 5 min to emit a beep from the piezo buzzer. Deployment delay mode (4) is used when the Kinematic Logger has been configured with settings ready for field deployment and memory has been erased ready for logging. This mode enables a delay in minutes to be set so that the Kinematic Logger can be installed inside a stone then transported to a field site without logging motion during the journey and deployment. Usually Kinematic Loggers are configured and installed in stones 1–2 days before field deployment, then are placed on a riverbed before a flood is expected. After the delay countdown timer expires, the Kinematic Logger enters logging mode (5). Logging mode is where the Kinematic Logger waits for a ‘significant motion’ interrupt from the IMU, upon which it wakes up and commences logging until a ‘no motion’ interrupt is received. The thresholds and durations for the motion and no motion interrupts are configurable when writing the settings to the Kinematic Logger (see Section 2.5). After the memory on the Kinematic Logger has been filled, or a maximum deployment timer has expired, the Kinematic Logger enters recovery mode (6). In this mode, the Kinematic Logger periodically wakes up to send LoRa recovery messages. The interval between messages, as well as the days of the week and hours of the day that the messages are sent are also user configurable (see Section 2.5). This minimises wasted battery power by not sending recovery messages at night or outside of feasible recovery times (see Section 2.6). This functionality is another benefit of using a real time clock.

### 2.4. High Frequency LoRa Recovery Messages

After field deployments, Kinematic Loggers are found by team members using directional antennas. To make this technique practical, frequent transmissions from each Kinematic Logger are needed. However, if the Kinematic Logger was constantly sending messages at a high frequency when in recovery mode it would rapidly drain the battery. To overcome this problem the Kinematic Logger is configured to listen for external commands for a defined time after each transmission. In practice this occurs as: (1) the Kinematic Logger transmits a LoRa message with its unique ID number (usually every 30 s when in recovery mode); (2) this message is received with a relay unit by a field team member; (3) the Kinematic Logger listens for a defined time after each transmission (usually 5 s); (4) the field team member sends a message from the relay unit with the unique ID number of the Kinematic Logger and the command to trigger high frequency transmissions; (5) the Kinematic Logger receives the message and commences high frequency transmissions (usually LoRa messages at 3 s intervals for 2 min). If the Kinematic Logger is not located within the 2 min period, then this process is repeated. This functionality balances the power consumption of frequent transmissions for recovery against limited battery capacity.

### 2.5. Software for Configuring Kinematic Loggers and Downloading Data

**Figure 2:**
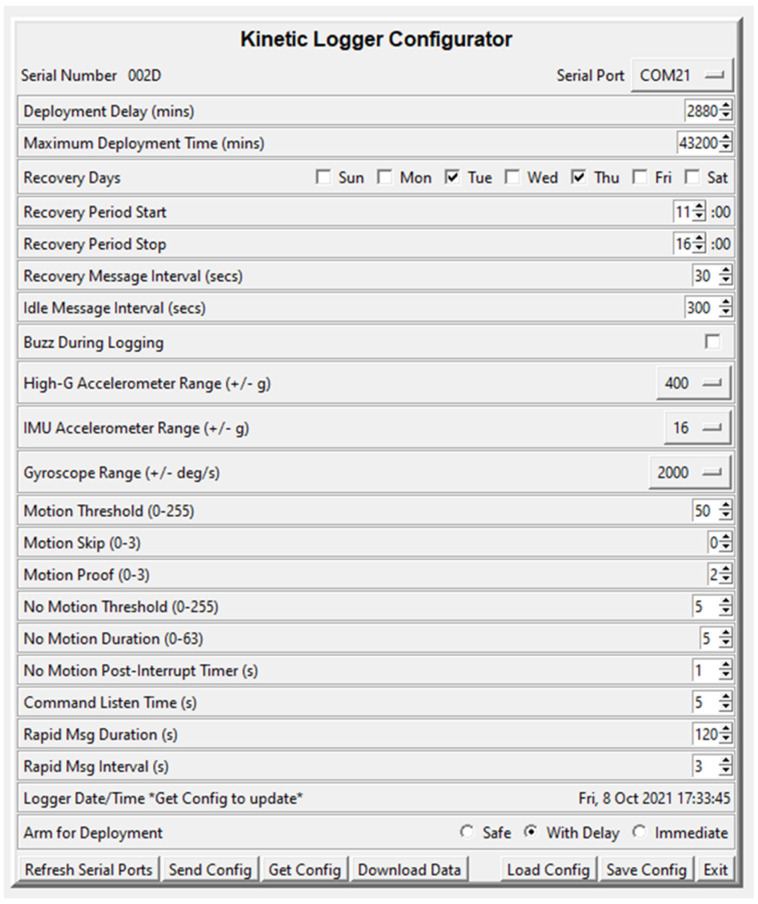
Kinematic Logger Configurator python application for setting deployment parameters, sending configuration, updating real time clock, and downloading data.

A python application was developed to set the configuration parameters on each Kinematic Logger before field deployment (Figure 2). This enables the deployment time parameters, recovery message parameters, measurement range, motion thresholds, and rapid message parameters to be easily set. The configuration can also be saved, enabling many Kinematic Loggers to be easily configured with the same settings. The configuration application is also used for downloading data from each Kinematic Logger after a field deployment, with an additional python script used for converting the encoded data to a .csv file for post-processing in MATLAB.

### 2.6. Power Consumption and Battery Life

The ESP32 microprocessor in the Kinematic Logger has a phase-locked loop module that can change (under software control) the base 40 MHz crystal frequency over a range from 10 MHz to 240 MHz. The faster the CPU speed, the greater the current consumption, so in order to minimise power the CPU speed is set within each run mode to the minimum required. The more active run modes require higher clock speeds to support communication with external devices (log memory, LoRa module, sensors). The CPU clock speeds, average current demands, and current consumption for each mode/function are summarized in Table 1.

In order to effectively plan field deployments, mode durations and LoRa TX/RX parameters it is essential to quantify total Current Consumption $CC_{T}$ (mAh), which can be found as the sum of Current Consumption in Deployment Delay Mode ($CC_{DDM}$), Logging Mode ($CC_{LM}$) and Recovery Mode ($CC_{RM})$. From Table 1, these equations are:

(1) $$CC_{T}=CC_{DDM}+CC_{LM}+CC_{RM}$$

(2) $$CC_{DDM}=T_{DDM}\times 0.175$$

(3) $$CC_{LM}=T_{LM}\times 0.195+84.2$$

(4) $$CC_{RM}=T_{RM}\times \left(0.175+f_{L,RM}\times CC_{L,TX}+f_{L,RM}\times 30\times T_{L,RX}\right)$$

where $T_{DDM}$ is the time in Deployment Delay Mode (hours), $T_{LM}$ is the time in Logging Mode (hours), $T_{RM}$ is the time in Recovery Mode (hours), $f_{L,RM}$ is the average frequency of LoRa transmissions in Recovery Mode (transmission per hour), $CC_{L,TX}$ is the current consumption of the LoRa transmission (at 14 dBm or 17 dBm), and $T_{L,RX}$ is the receiving/listening time after each LoRa transmission (hours).

If LoRa messages are also sent periodically in all modes (Figure 2—Idle Message Interval), which is useful for locating KL units outside of designated recovery times, the current consumption equations become:

(5) $$CC_{DDM}=T_{DDM}\times \left(0.175+f_{L,I}\times CC_{L,TX}+f_{L,I}\times 30\times T_{L,RX}\right)$$

(6) $$CC_{LM}=T_{LM}\times \left(0.195+f_{L,I}\times CC_{L,TX}+f_{L,I}\times 30\times T_{L,RX}\right)+84.2$$

(7) $$CC_{RM}=T_{RM}\times \left(0.175+f_{L,RM}\times CC_{L,TX}+f_{L,RM}\times 30\times T_{L,RX}+f_{L,I}\times CC_{L,TX}+f_{L,I}\times 30\times T_{L,RX}\right)$$

where $f_{L,I}$ is the frequency of LoRa transmissions (transmission per hour) in Idle Mode.

The equations for total current consumption can then be rearranged to solve for maximum recovery time ($T_{RM}$) for a planned deployment based on battery capacity and Kinematic Logger configuration. The Fanso ER14505M Lithium Thionyl Chloride batteries have a nominal capacity of 2200 mAh, with a functional capacity of 1760 mAh (using a conservative estimate of 80% of nominal capacity). Thus, the maximum recovery time is:

(8) $$T_{RM}=\frac{1760\;\mathrm{mAh}-CC_{DDM}-CC_{LM}}{\left(0.175+f_{L,RM}\times CC_{L,TX}+f_{L,RM}\times 30\times T_{L,RX}+f_{L,I}\times CC_{L,TX}+f_{L,I}\times 30\times T_{L,RX}\right)}$$

For a typical deployment, Deployment Delay Mode time ($T_{DDM}$) is 2 days (48 h). Logging Mode time ($T_{LM}$) is 60 days (1440 h). LoRa transmission power is 17 dBm. Idle messages are sent once per hour ($f_{L,I}$). LoRa transmission days in Recovery Mode are Tuesdays and Thursdays, with transmission times between 10 am and 4 pm, and LoRa transmission interval of 30 s, yielding an average hourly transmission frequency ($f_{L,RM}$) of 8.571 messages per hour. With LoRa listening time ($T_{L,RX}$) of 5 s (0.00139 h) after each transmitted message, this yields a maximum time in Recovery Mode $T_{RM}$ of 1764 h, or 73 days.

If no LoRa messages are sent or received the maximum Recovery Mode time is 9153.6 h (381.4 days), which is approximately 12 months. Thus, the main source of power consumption is LoRa TX and RX, with tuning of these deployment parameters crucial for establishing a sufficient maximum deployment time, while providing enough transmissions to find the sensors. With the typical deployment configuration listed above, total deployment times of at least 135 days (>4 months) are achieved.

### 2.7. Magnetometer Calibration and Sensor Fusion for Orientation

Post-processing of data from the Kinematic Logger is performed in MATLAB using the ‘Sensor fusion and tracking toolbox’. Data are extracted from the .csv file, converted from 16 bit integers into real world units and structured into ‘chunks’ based on each motion event, with real world timestamps generated. Data from the IMU magnetometer are then checked for any spikes, which occur very rarely (less than one point per million) but need to be identified and replaced before further processing. Spikes are detected with a 9-point moving median filter on magnetic field magnitude, with points that are more than 10× the moving median labelled as spikes and replaced with the median of the neighbouring good data for each axis. Calibration of the despiked magnetometer data is then performed using the magcal function in MATLAB. This step is critical to remove hard iron and soft iron disturbances to the magnetic field data due to interference from components on the circuit board. To obtain a good calibration, the Kinematic Logger should have turned through a wide range of angles at the site where measurements occurred. This is easily achieved if active transport of the sensors and rolling occurs during a flood. It can also be achieved by manually rotating the Kinematic Logger before deployment. Magnetometer (compass) calibration is critical for any device with a 9-axis IMU sensor and is not unique to the Kinematic Logger, for example: UAVs/drones (where it is usually performed manually) and cell phones (where it is usually performed automatically as they turn through a wide range of angles during typical use). Calibrated magnetometer data form a sphere and can then be scaled to match the magnitude of the local magnetic field.

To check the accuracy of calibrated magnetometer data, a Kinematic Logger was placed into different known orientations relative to the local magnetic field. The local magnetic field was obtained from the International Real-time Magnetic Observatory Network (intermagnet.org) and the Institute of Geological and Nuclear Sciences (GNS) at their Eyrewell monitoring site. In East North Up (ENU) format, the local magnetic field during testing was approximately 8410 nT, 19,200 nT, and 53,384 nT. The calibrated magnetometer data from the Kinematic Logger matched the reference data well. However, viewing the 3-axis magnetometer data illustrates the challenges of using magnetometer data for heading further from the equator. For example, a traditional compass is balanced on a pivot and held flat, thus only registering the East and North magnetic field vector components, with ‘magnetic North’ being the direction of the ‘net’ magnetic field from both the East and North components, which is easily corrected to ‘true North’ from published magnetic declination values based on geographic location. However, at 43° S (Christchurch, New Zealand) by far the largest component of the magnetic field vector points vertically, with the direction of gravity (down) needing to be estimated from the accelerometer data to remove the vertical component of magnetometer data and estimate the direction of magnetic North orthogonal to the gravity vector [34]. These estimates of absolute orientation for the Kinematic Logger are performed using the ahrsfilter function in MATLAB to fuse the 9-axis IMU data using a complementary indirect Kalman filter [35]. Tuning the complementary indirect Kalman filter to account for sensor noise and drift is necessary to obtain good convergence to real world orientation. Orientation quaternions are then used for Kinematic Logger orientation in real world coordinates or to rotate vector data from the Kinematic Logger frame of reference to the real-world frame of reference (or vice versa). For reliable orientation data it is recommended to deploy stones and Kinematic Loggers in natural rivers away from sources of magnetic interference, such as high-tension power lines or metal structures.

## 3. Stone Collection, Drilling, and Preparation for Installation of Kinematic Loggers

**Figure 3:**
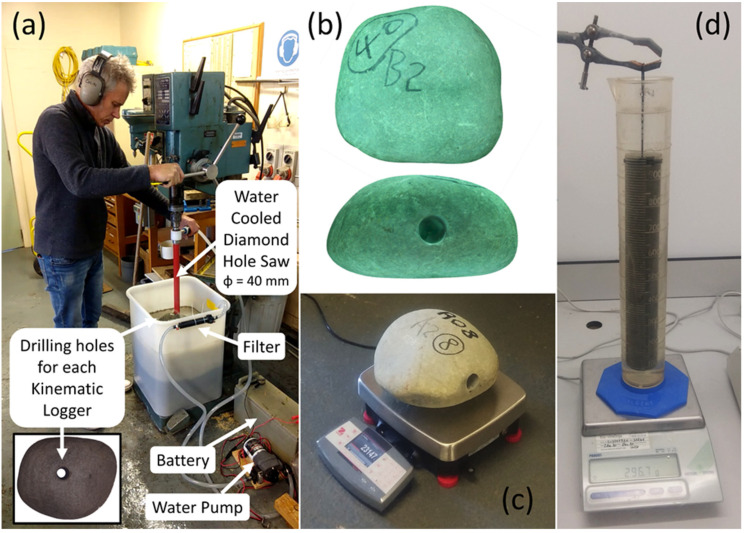
(**a**) NIWA technician Julian Sykes drilling holes through greywacke stones for installation of Kinematic Loggers, (**b**) 3D scans of the greywacke stones, (**c**) weighing stones before installation of Kinematic Loggers and field deployment, (**d**) measurement of volume of greywacke cores using Archimedes’ principle for calculations of greywacke density.

Greywacke stones from the Rangitata River were used for the ‘Rolling Stones’ project. Holes were drilled through the stones with a water-cooled diamond hole saw (Figure 3a) to house each of the Kinematic Loggers. The stones were placed in a large PVC tub with gravel in the bottom to stabilize them during drilling. A recirculating pump and filter system fed water through the centre of the diamond hole saw to cool the tip and remove greywacke cuttings. In total, 47 stones were drilled, covering a range of sizes (from 20 to 55 kg) and approximate shapes (i.e., spherical, disk, and rod). After stones were drilled, they were 3D scanned with an Einscan Pro HD structured light scanner. This was performed to obtain a 3D model of the stone surface before field deployment (Figure 3b), with 3D scanning repeated after field deployment to investigate surface abrasion and shape changes during bedload transport. Following 3D scanning the stones were weighed (Figure 3c) on a high precision benchtop scale (OHAUS Ranger 7000 R71MD60) with maximum weight of 60 kg, resolution of 1 g, and internal temperature compensation. To determine the density of the greywacke stones, the drilled cores were weighed on a high precision lab scale (METTLER TOLEDO PB8001), with maximum weight of 8100 g and resolution of 0.1 g. The volume of the cores was determined using Archimedes’ principle by weighing the displaced water when freely suspending the core within a measuring cylinder with the core fully submerged (Figure 3d). There was little variation between the density of the greywacke cores (likely because the stones were collected from the same location) and measurements were ceased after processing five randomly selected cores, which had an average density of 2726.270 kg/m^3^ and standard deviation of 4.098 kg/m^3^.

## 4. Sensor Housings, Kinematic Logger Installation Orientation, and Recovery Systems

### 4.1. Sensor Housings

**Figure 4:**
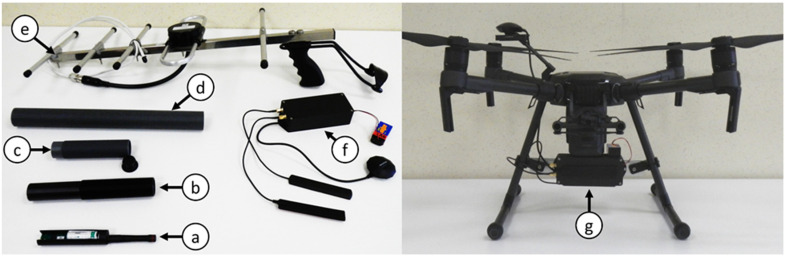
(**a**) Kinematic Logger, battery, and 433 MHz antenna installed in a 3D printed sleeve, (**b**) threaded waterproof housing for the Kinematic Logger, (**c**) threaded end plugs to span the distance between the Kinematic Logger and the surface of the stone, (**d**) threaded sleeve that is glued into the stone, (**e**) directional Yagi antenna with handle and wrist support for Kinematic Logger recovery missions, (**f**) relay unit with antennas and 9 V battery, (**g**) relay unit installed on a DJI M210 UAV.

Sensor housings were designed in Autodesk Inventor to support the Kinematic Logger PCB and provide a durable waterproof enclosure for field deployment in flooded rivers. A chassis with a slotted groove to hold the PCB was designed and 3D printed on a Markforged X7 from Onyx material (Figure 4a). This was then inserted into a durable waterproof housing (Figure 4b) that was fabricated out of acetyl and featured a waterproof end cap with an o-ring seal. Since the drilled holes through the stones were variable length (and longer than the durable waterproof housing) extra end plugs were also fabricated (Figure 4c). These were made from PVC and were cut to length for each stone. To enable the Kinematic Logger housings and end plugs to be easily installed and removed from stones, threaded PVC sleeves (Figure 4d) were also fabricated. These were permanently glued into the stones using ‘Tarzan’s Grip’ glue and enabled the threaded Kinematic Logger housings and end plugs to be screwed into place. Custom tools were made for installing the Kinematic Logger housings and end plugs, that fitted into a slotted groove or into two holes that were drilled in their ends. The minimum stone diameter (i.e., drilled axis length) for installation of kinematic loggers and housings is approximately 240 mm, with an ideal length of approximately 300 mm (to allow space for end plugs).

### 4.2. Ballasting Sensor Housings

One challenge with installing sensors and housing in the centre of the stones is to keep the mechanical characteristics of the stone, and therefore its motion, unaffected. To achieve this the cylindrical core of rock that was removed during drilling would need to be replaced with another core that has the same: mass, centre of mass, and rotational inertia. Originally it was planned to ballast the end plugs with tungsten carbide, which has a high density of 15.6 g/cm^3^ and is non-toxic, being commonly used for making tools and jewelry. Tungsten carbide grits were sourced, mixed into a slurry with epoxy, and set into hollow end plugs (Figure 4c). Although this approach was successful at correcting the mass and centre of mass, it was very challenging to obtain a mass distribution that would correct rotational inertia around all three of the axes. Although it was easy to plan a suitable mass distribution and calculate the required epoxy to tungsten carbide ratios and layers, actually fabricating such an end plug was very difficult. The choice of tungsten carbide as ballast is good from a density and environmental impact perspective (compared to lead for example), however the extreme hardness of tungsten carbide means that it is challenging to work with after being set in epoxy (e.g., it cannot be cut or trimmed without damaging saw blades). Another problem with using ballasted end plugs was that they had a minimum feasible length that would provide the correct ballast (even just for mass and centre of mass), meaning that stone core lengths (i.e., stone diameters) had to be approximately 360 to 400 mm, resulting in the deployment of very large stones (boulders) between 40 kg and 55 kg. Even with two team members using a specially built stone carrying stretcher, these stones are challenging to transport around field sites. Stones of this size also require more extreme conditions to initiate motion, making data recording less likely in medium size floods. Because of these issues, the use of tungsten carbide ballasted end plugs was abandoned. Instead, the PVC end plugs were cut to shorter lengths and used to span smaller distances from the threaded waterproof housing to the surface of the stone and were then trimmed flush with the surface using a Makita multi-tool with a reciprocating saw head. This enabled more manageable stones between 20 kg and 40 kg to be deployed, with the differences in mass, centre of mass, and rotational inertia quantified and provided as sources of uncertainty in the results. For example, the addition of unballasted sensors typically resulted in reduction in the mass of the stone of less than 2%, negligible changes in rotational inertia around the drilled axis, and minor changes in rotational inertia around the axes orthogonal to the drilled axis. If using Kinematic Loggers to study rockfall or landslide dynamics it may be worth ballasting the end plugs, however for bedload transport in rivers it is not practical.

### 4.3. Relay Units and Recovery Systems

**Figure 5:**
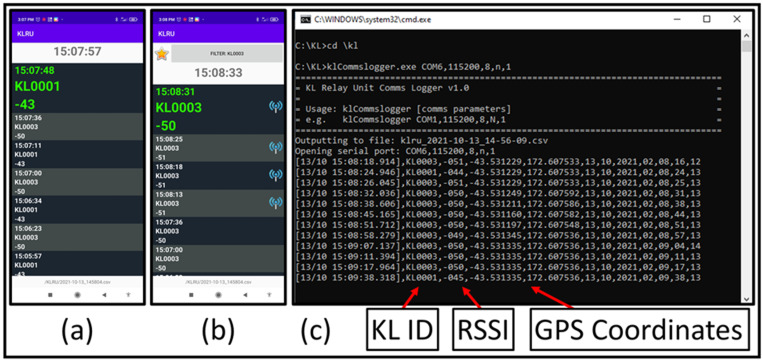
(**a**) KLRU android application for viewing LoRa messages with Kinematic Logger ID number and RSSI, (**b**) KLRU application in hunting mode where LoRa messages from other sensors are hidden, and reply messages are automatically transmitted to the targeted sensor to trigger rapid communications, (**c**) python script on the base station computer logging relayed LoRa messages and the GPS coordinates of the relay unit to a .csv file for processing.

Kinematic Loggers are re-located based on Received Signal Strength Indicator (RSSI) data from transmitted LoRa messages. To achieve this required development of novel hardware, firmware, software, and recovery methods. A dual frequency relay unit was developed that can send/receive 433 MHz LoRa messages to/from Kinematic Loggers, then relay these messages at 915 MHz back to a base station computer for logging as a .csv file and interpretation. Each relay unit (Figure 4f) consisted of: (1) an Arduino Due board (which supports dual serial communications for simultaneous listening at 433 MHz and re-transmission at 915 MHz); (2) a carrier PCB containing a 433 MHz LoRa Radio Module (HopeRF RFM98W) and a 915 MHz LoRa Radio Module (HopeRF RFM95W); (3) a 433 MHz antenna (RF Solutions ANT-433IBAR3-SMA) for communications with the Kinematic Loggers; (4) a 915 MHz antenna (LINX ANT-8/9-VDP-2000-SMA) for communications with the base station computer; (5) a GPS receiver (GPM-808G) for adding the location of the relay unit to the relayed messages; (6) a 3D printed case (printed on a Markforged X7 from Onyx material with layers of continuous carbon fibre); and (7) a battery (i.e., 9 V lithium battery, or two 18650 batteries connected in series). The relay units can also be connected to an android cellphone via a USB cable, with a custom android application developed to visualise RSSI (Figure 5a), log LoRa messages to a .csv file on the phone, and communicate with the Kinematic Loggers to trigger rapid communications (Figure 5b). The relay units are multifunction and can be run in 3 operation modes (i.e., independent relay unit (433 MHz RX, 915MHz TX), cellphone relay unit (433 MHz TX/RX, 915 MHz TX), or computer receiver unit (915 MHz RX), with a jumper on the carrier board used to switch between modes. The multiple functionalities of the relay units provide flexibility and redundancy, with the six relay units that were manufactured being rapidly interchangeable.

**Figure 6:**
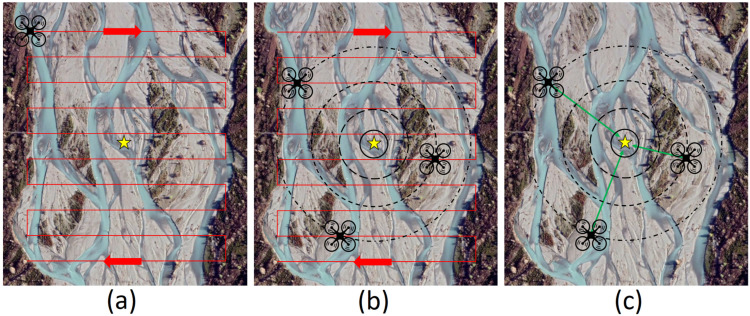
(**a**) Zigzag flight path of UAV with relay unit over the river to locate the deployed stone and Kinematic Logger (yellow star), (**b**) the UAV receives LoRa messages from the Kinematic Logger at multiple locations along the flight path, (**c**) the location of the Kinematic Logger is estimated from received signal strength and triangulation principles.

During field recovery missions one of the relay units is mounted on a DJI M210 UAV (Figure 4g) and is flown over the riverbed in a zigzag pattern (Figure 6), relaying any Kinematic Logger messages to the base station computer where they are logged to a .csv file (Figure 5c). The relayed messages contain: Kinematic Logger unique ID, RSSI, GPS coordinates of the relay unit, and timestamp. Once the UAV has completed its zigzag survey mission, the .csv file of data is analysed using a pre-compiled MATLAB script and generates a map of Kinematic Logger unique IDs and RSSI throughout the surveyed area (see Section 4.4). After user interaction with the map and location optimisation, a .csv file of estimated coordinates for all the Kinematic Loggers is output. This .csv file is then copied via USB from the base station computer to the RTK GPS handheld unit (Trimble TSC3). Team members set out from the base station in a 4WD vehicle (e.g., Toyota Hilux or Toyota Landcruiser) with the RTK GPS unit in stakeout mode to provide navigation to the general location of each Kinematic Logger. To pinpoint and recover Kinematic Loggers, the team bring a relay unit, cell phone and directional Yagi antenna (Figure 4e), shovels for digging up buried Kinematic Loggers and a waterproof metal detector (Minelab SDC2300) to assist with locating any buried or underwater Kinematic Loggers. Once the Kinematic Logger and host stone are located, they are carried back to the vehicle on a custom-made stone carrying stretcher. Field tests of the recovery systems and information on time taken to find Kinematic Loggers are provided in Section 5.

The recommended altitude for flying the Relay Unit on the UAV and flight line spacing will be mission dependent, however as a rule of thumb for dry Kinematic Loggers 20 m altitude and 20 m flight line spacing is recommended. For underwater Kinematic Loggers 5 m altitude (if possible) with 5 m flight line spacing is recommended. Reliably receiving signals from underwater Kinematic Loggers requires closer flight lines, lower altitudes, and slower flight speeds, since the Relay Unit should ideally be directly above the Kinematic Logger when it transmits recovery messages, to minimise the signal path length through water (and associated attenuation). LoRa messages from underwater Kinematic Loggers can generally be received from a depth of 1.5 m (during tests in New Zealand rivers), however they cannot be easily triangulated due to: depth and angle dependent path losses, refraction at the air–water interface, and antenna orientations relative to the water surface. Where a signal from an underwater Kinematic Logger is received, it is easiest to walk to the location of maximum signal strength then pinpoint it with a directional antenna and metal detector.

### 4.4. Signal Strength Analysis

The Received Signal Strength Indicator (RSSI) is a useful proxy for the power of a received signal. Although RSSI is not a standardised parameter and varies with manufacturer hardware, it can still be used to roughly estimate distance. For idealised isotropic antennas, the signal will spread out in a spherical shell and decrease in power with the inverse square of distance. However, in reality, antennas are not isotropic and there will be additional attenuation of radio signals as they travel, depending on factors such as the transmission frequency and physical characteristics of the medium of propagation (notably conductivity). For example, radio transmissions in sea water are attenuated far more quickly than those in freshwater due to conductivity [36]. For deployments in New Zealand rivers, field tests were performed that showed that LoRa transmissions could be received from 1.5 m deep under freshwater. Field tests showed little attenuation of signals due to placing Kinematic Loggers inside rocks, or when they were buried in dry substrate comprised of cobbles, gravel, sand, and silt. Tests were not performed with Kinematic Loggers buried under a clay layer, and this may cause more attenuation than the sediment mixtures that are common in New Zealand braided rivers.

For signal transmission through one medium the approximate relationship between RSSI (in dBm) and transmission distance is $RSSI\approx -\left(10\times n\times \log_{10}\left(d\right)-A\right)$, where A is RSSI at 1 m reference distance, d is transmission distance, and n is the loss parameter (or loss exponent) for the specific environment [37]. Although this relationship assumes antennas with isotropic gain, and an isotropic transmission environment, it still provides a good fit to empirical data of RSSI and distance. In MATLAB this is easily implemented as a least squares fit of the simplified equation $RSSI\approx a\times \log_{10}\left(d\right)+b$ where RSSI and d are input data from field measurements, while both a and b are fitted constants. To estimate distance from RSSI the equation is then rearranged as $d\approx 10^{\left(\frac{RSSI-b}{a}\right)}$. For the calibration data with our LoRa transmitters and antennas *a* = −10.48 and *b* = −39.68, with *R*^2^ = 0.8789. The value of *R^2^* reflects some slight scatter in the data, which is to be expected due to variations in the relative orientations of the transmission and receiving antennas (and thus gain) during collection of calibration data. This relationship was found to be applicable for Kinematic Loggers on the dry surface of the riverbed or buried in dry (or slightly damp) substrate. For underwater Kinematic Loggers signal attenuation is much more significant, and a slightly different recovery strategy is needed (as discussed in Section 4.3).

**Figure 7:**
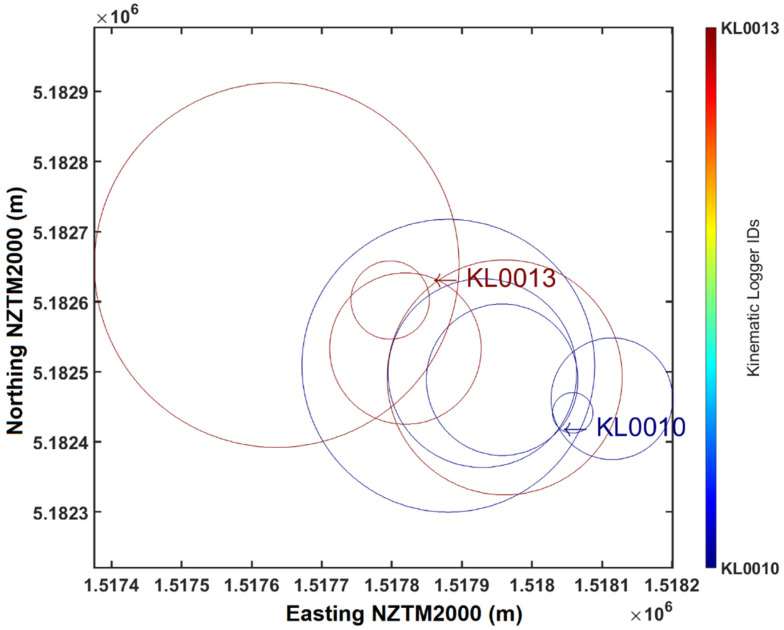
Field data from the Selwyn River showing received LoRa messages from Kinematic Loggers KL0010 and KL0013. The intervals between axis tick marks is 100 m. Circle centres are the drone locations when a LoRa message was received. Circle radius is derived from the calibration equation for RSSI and distance. The intersection between circles (tip of the label arrows) are the starting locations for the ground based search for KLs.

By flying the relay unit in a zigzag pattern over the deployment site, it is possible to obtain data on RSSI, Kinematic Logger unique identifier, and relay unit GPS location across the deployment site (Figure 6). These data are then interpreted with a pre-compiled MATLAB executable to map and triangulate the approximate locations of the sensors, providing starting locations for ground based recovery with directional antennas and a metal detector. The most practical method for data interpretation in the field was found to be plotting a map of overlapping distance estimation circles based on RSSI for all of the Kinematic Loggers (Figure 7). These circles converge at the approximate location of each Kinematic Logger, which is where field teams are deployed to commence searching. This approach was preferred to a true triangulation method, since it is not sensitive to antenna orientations (both TX and RX), does not require data on drone altitude, can be easily visualised, and allows the drone to be flown down to low altitude to search for a Kinematic Logger (particularly those underwater), providing tighter estimation circles. The maps of Kinematic Logger locations have overlaid labels of where the maximum RSSI for each Kinematic Logger was observed. These labels are interactive and can be dragged by the user to the optimal location for searching to commence. User interpretation and interactivity are particularly advantageous if Kinematic Loggers are underwater and signals are being attenuated, in which case the starting location for ground searching can be set to the location where the strongest signal was received from an underwater Kinematic Logger (as discussed in Section 4.3). Following location optimisation, a .csv file of Kinematic Logger IDs and starting GPS coordinates is produced. This is copied by USB from the home base laptop, to the RTK GPS Trimble TSC3 handheld unit and field team members are deployed with the unit in ‘stakeout’ mode to find the Kinematic Logger.

### 4.5. Kinematic Logger Installation Orientation Relative to the Stone

**Figure 8:**
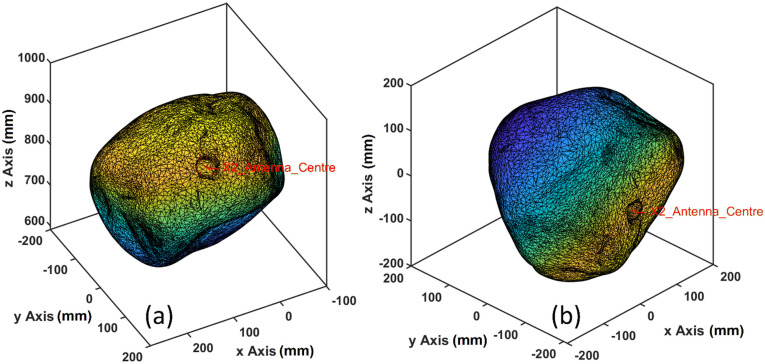
(**a**) Stone point cloud and mesh with arbitrary origin and orientation after 3D scanning, (**b**) stone point cloud and mesh with origin and orientation matching that of the IMU.

To investigate stone dynamics, it is critical to relate the origin and orientation of the Kinematic Logger IMU to the origin and orientation of the stone it is installed within. The point cloud data and mesh from the 3D scan is in a coordinate system with an arbitrary origin and orientation, so it needs to be translated and rotated to match the origin and orientation of the IMU. When the Kinematic Logger is installed inside the stone it is screwed into place, creating challenges for determining its orientation relative to the stone. The origin of the IMU can be easily measured from offsets to the stone surface, and the Y axis direction of the IMU can also be easily determined (since it points in the same direction as a unit vector through the centre of the drilled hole). However, determining the orientation of the X and Z axes relative to the stone is more challenging. To achieve this a calibration procedure was developed where the stone is placed on a pivot through its central axis, with the Kinematic Logger recording accelerometer data. The stone will then rotate until its centre of mass is below the pivot axis. By recording acceleration data (which sum to 1 g when stably balanced) the direction of the centre of mass in the IMU frame of reference can be found. It is then possible to find the centre of mass of the stone relative to the central drilled axis from the 3D scan data in the stone frame of reference. By combining these data and measurements of offsets to the IMU origin, the stone point cloud and mesh can be translated and rotated to match the IMU frame of reference (Figure 8). These data are essential for calculating mass properties of the stone (i.e., centre of mass and rotational inertia) in the IMU frame of reference, which are needed for interpreting IMU data to investigate surface forces, angular velocities, torque, and angular accelerations. The processing steps were: (1) import the rock 3D scan file (.stl format) to MATLAB; (2) decimate the mesh and point cloud (the very high resolution scan data are only needed for investigation of surface abrasion by comparing the rocks’ geometry before and after their deployment in rivers); (3) select points around the rim of the drilled holes; (4) fit an ellipse through the 3D points and find the centre using functions from the ‘Object-oriented tools for fitting conics and quadrics’ developed by [38]; (5) translate the point cloud and hole centres so that the origin is now at the antenna end of the drilled hole; (6) compute the unit vector along the drilled hole axis (i.e., between the two ellipse centres); (7) find a unit quaternion from the hole axis unit vector, to the Y axis unit vector, then rotate the point cloud and hole centre points to match (i.e., so the stone Y axis now matches the IMU Y axis); (8) translate the point cloud along the Y axis based on calibration measurements of the distance from the stone surface to the IMU origin; (9) find the centre of mass of the stone and create a unit vector orthogonal to the Y axis that points in the direction of the centre of mass (i.e., in the XZ plane); (10) from the IMU calibration data find the X and Z accelerations when the stone is balanced on the pivot (i.e., such that the centre of mass is underneath the Y axis), then convert these to a unit vector; (11) find the unit quaternion between the unit vectors from steps 9 and 10, then rotate the point cloud and mesh so that the stone frame of reference matches the IMU frame of reference; (12) save the rotated and translate stone as a .stl file; (13) load the .stl file into Solidworks as a solid body; (14) draw a 40 mm diameter hole on the XZ plane centred on the origin; (15) perform an extruded cut to complete the hole through the centre of the stone; (16) set the material density to that of greywacke stone (with the measured density from Section 3); and (17) compute the mass properties of the stone (i.e., mass, volume, centre of mass, and rotational inertia).

## 5. Field Tests of Kinematic Logger Recovery Systems

**Figure 9:**
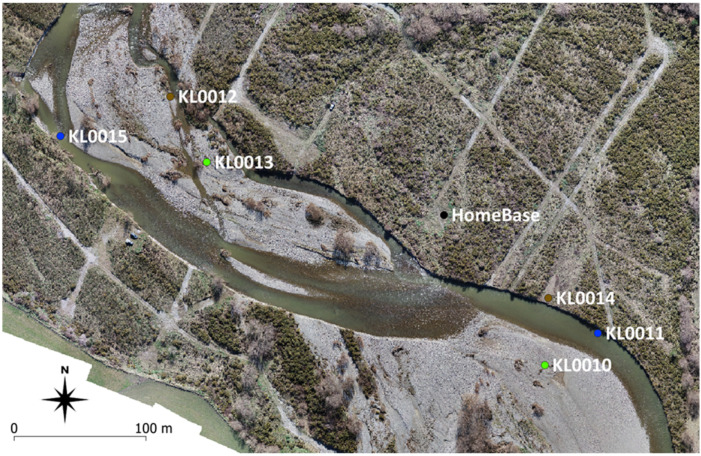
Deployment locations of Kinematic Logger units during field recovery tests. Green dots are units located on the surface, brown dots are buried units, and blue dots are underwater units.

To provide an accurate real-world test of Kinematic Logger recovery systems, a ‘single blind’ field deployment and recovery test was performed. The test was undertaken in the Selwyn River, New Zealand (−43.50491, 171.98332) by two teams. Team #1 hid the Kinematic Loggers within a 600 m long by 200 m wide reach of the river, with the location of each hidden stone and Kinematic Logger surveyed with RTK GPS. Team #2 then assembled the M210 UAV, relay units, directional antennas, metal detector, and logging computer, then commenced searching for the hidden Kinematic Loggers, with the time taken to find each unit recorded. Equipment setup takes approximately 1 h, with setting up the RTK GPS system being the most time consuming. The test was designed to be as close to real world conditions as possible, with some stones deployed underwater and others buried (Figure 9). The RTK GPS base station location formed the home base for setting up the recovery system equipment and was located in an area of dense riparian vegetation that blocked the field of view of Team #2 upstream or downstream, so they had no prior knowledge about the location of the hidden stones and Kinematic Loggers. The test also provided valuable information about the required transmission signal strength to find the hidden Kinematic Loggers (14 dBm or 17 dBm), which is critical information for planning effective large scale deployment during floods, where using excessive transmission power will waste battery capacity, while insufficient transmission power will make it more likely to lose Kinematic Loggers. Six Kinematic Loggers were deployed (Figure 9), with two units on the surface, two units buried, and two units underwater (Table 2). Three of the units were configured to transmit LoRa messages at 17 dBm, while the remaining three units transmitted at 14 dBm. The time taken to recover the Kinematic Logger units was measured from the commencement of UAV flights. The UAV was flown at 50 m altitude in a zigzag pattern over the study site to provide starting locations for the ground-based search with directional antennas.

All six of the Kinematic Logger (KL) units were recovered with the directional antennas (Table 2). However, KL0015 was not detected by the UAV during the initial mapping flights. The deployment of KL0015 provided useful information about the functional limitations of detection (i.e., worst-case scenario) under 1.15 m of water with low transmission power of 14 dBm. After drone flights were completed and KL0015 was not detected, Team #1 provided a hint to Team #2 that KL0015 was at the Northwest end of the study site. This provided sufficient information to become close enough to KL0015 that it was then detected with the directional antennas and recovered. There was negligible attenuation of signals from the buried KL units, and these were located with similar ease to the ones on the surface. The buried KL units were in soft sediment (i.e., sand and silt) so were not difficult to recover, however during real world deployments (i.e., floods) recovery of any buried units is expected to be more problematic in stony river substrate. The test provided valuable information about the feasibility of field recovery and information on deployment settings. For real world deployments of KL units, all sensors will be configured to transmit at 17 dBm and drone flights will occur at a lower altitude of ~20 m over land, and ~5 m over water to increase the probability of locating any underwater units. The time taken to recover the Kinematic Loggers indicates that it should be feasible to recover a full deployment of ~20 KL units in one day following a flood, provided that sufficient team members are available to help with locating and digging up buried units. Underwater units provide the greatest challenge for detection and recovery, thus we consider that when working in braided rivers it should be best to deploy units in braided river side channels that will likely dry up following floods rather than in perennially-wetted main braids.

## 6. Conclusions and Future Work

Extensive technical development was completed to develop rugged ‘Kinematic Loggers’ to measure stone dynamics. These sensors can record 1 h and 50 min of 9-axis IMU data at 400 Hz, as well as 3-axis high-g accelerometer data at 1000 Hz and can be deployed in extreme environments. Methods to drill holes through the stones, prepare stones for field deployments, and calibrate the origin and orientation of Kinematic Loggers within the stones were also developed. Novel relay units were developed for finding the Kinematic Loggers after field deployments, based on the received signal strength of LoRa radio transmissions. These relay units can be flown on UAVs, deployed with handheld directional antennas, or used for logging data to a base station computer. Custom Android, Python, and MATLAB applications were also developed for viewing, logging, and processing the relay unit data. The field test at the Selwyn River demonstrated the effectiveness of the recovery systems. Future work recording stone dynamics during bedload transport and data analysis is ongoing. These sensors and systems are designed for measurement of stone dynamics during floods but could also be deployed to measure landslides and rockfalls.

## Figures and Tables

**Table 1:** Kinematic Logger modes and functions with CPU frequency, average current, and current consumption.

Mode/Function	CPU Frequency When Awake	Average Current (mA)	Current Consumption (mAh)
Deployment delay	10 MHz	0.175	Delay duration × 0.175
Logging (awaiting motion interrupt)	10 MHz	0.195	Logging duration × 0.195
Logging (active)	160 MHz	46	1.83 h × 46 = 84.2
Recovery mode	10 MHz	0.175	Recovery duration × 0.175
LoRa Transmission 14 dBm	80 MHz	N/A	0.015
LoRa Transmission 17 dBm	80 MHz	N/A	0.017
LoRa Receiving	80 MHz	30	Listening time × 30

**Table 2:** Kinematic Loggers deployed during field recovery tests.

Kinematic Logger ID	LoRa Transmission Power	Deployment Environment	Depth (m)	Proximity to Home Base (m)	Time to Recovery the KL (min)	Detected with UAV
KL0010	17 dBm	Surface	0	137.1	102.5	Y
KL0011	17 dBm	Underwater	0.6	146.3	105.1	Y
KL0012	17 dBm	Buried	0.7	226.1	63.1	Y
KL0013	14 dBm	Surface	0	184.2	70.2	Y
KL0014	14 dBm	Buried	0.6	101.3	96.9	Y
KL0015	14 dBm	Underwater	1.15	295.8	77.4	N

## Data Availability

Data on Kinematic Loggers specifications, power consumption, signal strength relationships, and field tests are contained within this article. Any other complementary data related to the Kinematic Loggers or field tests can be made available upon request from the corresponding author.
